# Sonographic Features of Rectus Femoris Muscle in Patients with Metabolic Dysfunction-Associated Fatty Liver Disease and Their Correlation with Body Composition Parameters and Muscle Strength: Results of a Single-Center Cross-Sectional Study

**DOI:** 10.3390/biomedicines12081684

**Published:** 2024-07-28

**Authors:** Anna F. Sheptulina, Adel A. Yafarova, Elvira M. Mamutova, Oxana M. Drapkina

**Affiliations:** 1 Department of Fundamental and Applied Aspects of Obesity, National Medical Research Center for Therapy and Preventive Medicine, Moscow 101990, Russia; adeleyafaroff@gmail.com (A.A.Y.); emamutova@gnicpm.ru (E.M.M.); drapkina@bk.ru (O.M.D.); 2 Department of Therapy and Preventive Medicine, A.I. Evdokimov Moscow State University of Medicine and Dentistry, Moscow 127473, Russia

**Keywords:** ultrasound, skeletal muscles, dual-energy X-ray absorptiometry, bioimpedance analysis, metabolic dysfunction-associated steatotic liver disease

## Abstract

This study aimed to describe sonographic features of rectus femoris muscle (RFM) in patients with metabolic dysfunction-associated fatty liver disease (MASLD) and their correlation with body composition parameters and muscle strength. A total of 67 patients with MASLD underwent dual-energy X-ray absorptiometry (DEXA), bioimpedance analysis (BIA), muscle strength measurement (grip strength [GS] and chair stand test [CST]), and ultrasound (US) investigation of the RFM in the dominant thigh using a 4 to 18 MHz linear probe. MASLD patients exhibited increased RFM echogenicity, possibly due to fatty infiltration. We confirmed that the greater the subcutaneous fat thickness, the smaller was the muscle mass (*p* < 0.001), and the lower was the muscle strength (*p* < 0.001 for GS and *p* = 0.002 for CST). On the contrary, the greater the anteroposterior diameter (APD) of RFM, the higher was the muscle mass (*p* < 0.001), and the greater was the muscle strength (*p* < 0.001 for GS and *p* = 0.007 for CST). In addition, APD of the RFM and stiffness of RFM exhibited direct correlation with bone mineral density values of the lumbar spine (*p* = 0.005 for both GS and CST). We concluded that US investigation of the RFM in the dominant thigh can be helpful in identifying MASLD patients at a high risk of musculoskeletal disorders given repeated point-of-care clinical evaluations.

## 1. Introduction

In 2023, a Multisociety Delphi Consensus Statement on New Fatty Liver Disease Nomenclature was published, proposing to rename non-alcoholic fatty liver disease (NAFLD) to metabolic dysfunction-associated steatotic liver disease (MASLD) [1]. This novel term is thought to better reflect the etiology and pathogenesis of MASLD, and its association with the diseases of other organs attributed to the lipid and/or glucose metabolism disturbances, insulin resistance, and ectopic fat accumulation as well. It also helps expand the range of possible markers of MASLD, targets for therapy, and factors influencing the natural history of the disease, its progression, and outcomes. Recently, it was shown that accumulation of ectopic fat in obesity may occur not only in the liver, but also in other internal organs or compartments of the body, including skeletal muscles. The latter are not originally intended for fat storage [2]. In 2021, M. Nachit et al. published a study conducted on 184 patients with MASLD and morbid obesity [3]. At inclusion in the study and 12 months later, a dietary intervention or a bariatric surgery patient underwent liver biopsy in order to evaluate MASLD activity, as well as computed tomography (CT) or bioimpedance analysis (BIA) to assess quantity and quality of the skeletal muscles. It was shown that absolute fat content in muscles (skeletal muscle fat index [SMFI]) calculated using the CT-based psoas muscle density) was significantly higher in patients with metabolic dysfunction-associated steatohepatitis (MASH) compared with those with conventional steatosis. Additionally, patients with MASH had lower muscle mass vs. those without it. Interestingly, the reduction of SMFI by ≥11% due to weight loss, measured by CT-based psoas muscle density, was associated with the resolution of MASH, albeit muscle mass remained unchanged.

Also, myosteatosis (fat infiltration into the muscles) is thought to contribute to sarcopenia, a condition characterized by gradual and generalized loss of muscle mass, strength, and function. Previously viewed as an aging-related phenomenon, sarcopenia may actually occur at any age due to various factors, such as insufficient physical activity, poor diet, and chronic noncommunicable diseases [4,5]. Sarcopenia is associated with adverse outcomes including reduced quality of life, physical disability, and mortality [6]. For instance, in a study by G. Kim et al. based on subjects with and without MASLD who were followed for seven years, it was established that an increase in the skeletal muscle mass could potentially decelerate, stop, or even reverse the progression of MASLD over time and help resolve existing MASLD [7].

Despite the progress in understanding the pathogenesis of MASLD and the significant amount of research aimed at developing new medications for its treatment, lifestyle modification, including increased physical activity, still remains the most effective method for combating this liver condition. Physical activity helps preserve a sufficient amount of muscles in the body and support their health, which, according to published evidence, is important in the prevention of MASLD and may contribute to its treatment [8,9].

Furthermore, the above-mentioned data confirm that the quality and quantity of muscles are critical prognostic factors affecting the likelihood of developing MASH and advanced liver fibrosis independently of the presence of obesity and insulin resistance [6,10]. Regular assessment of skeletal muscle mass quality and quantity constitutes a useful tool capable of identifying MASLD patients with reduced muscle mass and strength, who are already at increased risk for the progression of MASLD and for the development of its liver-specific and extrahepatic complications. This category of patients may derive the greatest benefits from measures aimed at preserving and increasing muscle mass and strength, especially considering that higher physical activity by itself is associated with a dose-dependent reduction in the risk of MASLD development and progress, regardless of dietary modifications [11].

According to the revised European consensus on definition and diagnosis of sarcopenia, tools recommended for the measurement of muscle mass and muscle quality include dual-energy X-ray absorptiometry (DEXA), BIA, CT, or magnetic resonance imaging (MRI) [12]. Ultrasound (US) investigation of skeletal muscles was also cited in this document in the Alternative or New Tests and Tools section and was characterized as a reliable, valid, inexpensive, and noninvasive technique that can be easily used in both inpatient and outpatient settings. Yet, the European Working Group on Sarcopenia in Older People (EWGSOP) concluded in 2019 that because the majority of studies concerning the diagnostic accuracy of the US imaging of the muscle mass for the detection of sarcopenia were performed on older patients, more research was needed to validate the predictive ability of this method for patients with different health conditions and varying functional status before the method could be recommended for routine clinical practice [13,14,15].

It was recently established that US imaging of the muscle mass may represent a dependable alternative to electromyography in detecting neuromuscular diseases in children [14,16]. The US imaging allows assessing not only echogenicity of the muscle, which increases with myosteatosis and/or muscle fibrosis, but also muscle thickness, which may be reduced in cases of muscle atrophy, as well as the degree of muscle vascularization suggesting the presence of inflammation. Moreover, changes in muscle architecture may also affect tissue elasticity and muscle anisotropy [17]. Taking into account these beneficial features and capabilities of the musculoskeletal US, we performed a study aimed at describing and classifying the sonographic features of skeletal muscles in patients with MASLD but without significant fibrosis. To the best of our knowledge, such studies have not yet been conducted among patients with MASLD.

## 2. Materials and Methods

### 2.1. Study Participants

We conducted a single-center cross-sectional study at the Clinical and Diagnostic Division of the National Medical Research Center for Therapy and Preventive Medicine. All eligible MASLD patients, seeking medical care at the Clinical and Diagnostic Division during July–November 2023, were consecutively and prospectively included in this study. The study was carried out in accordance with the Declaration of Helsinki guidelines. The study protocol was approved by the Research Ethics Committee of the institution (protocol # 01-03/20 of 23 February 2023). All included patients signed an informed consent form.

Inclusion criteria were as follows: (1) age of 18 to 70 years; (2) confirmed diagnosis of MASLD; and (3) absence of concomitant liver disease (based on serological testing for hepatitis B and hepatitis C viral infections, as well as serum γ-globulin, immunoglobulin G level, and serum ferritin concentration).

Exclusion criteria involved concomitant liver disease and significant alcohol consumption in the amount of >20 g/day as recognized by the Alcohol Use Disorders Identification Test (AUDIT), and patients were excluded if they had a score of ≥8. Other exclusion criteria included acute infections and pathological conditions. Obesity, exacerbation of chronic noncommunicable diseases (within four weeks before inclusion), history of any malignant neoplasms, advanced chronic kidney disease (GFR < 30 mL/min/1.73 m^2^), mental disorders, poor cooperation with study personnel, fractures of lower limbs within 6 months before the study with a persistent negative effect on the functional status, any clinically significant impairment or disease that interfered with motion and self-care, pregnancy and breastfeeding, and lack of the signed consent form.

Patient interview, collection of anthropometric data (weight, height, body mass index [BMI], waist circumference [WC], and hip circumference [HC]), laboratory tests, abdominal US, measurement of liver stiffness using point-shear wave elastography (C5-1 convex probe, Philips Affiniti 70, Philips, Amsterdam, The Netherlands), DEXA (Lunar iDXA, GE HealthCare, Chicago, IL, USA), BIA (ABC-02 Medass, STC Medass, Moscow, Russia), as well as US imaging of the rectus femoris muscle (RFM) in the dominant thigh (eL18-4 linear probe, Philips Affiniti 70, Philips, Amsterdam, The Netherlands) were performed during the same visit.

### 2.2. Diagnosis of Metabolic Dysfunction-Associated Steatotic Liver Disease

Diagnosis of MASLD was based on the criteria proposed by the 2023 Multisociety Delphi Consensus Statement on New Fatty Liver Disease Nomenclature, including the presence of (1) abdominal ultrasonography-based signs of liver steatosis, specifically brightness of liver parenchyma, liver-to-kidney contrast, decreased expression of hepatic vessels, or impaired visualization of deeper liver parenchyma and the diaphragm; and (2) at least one cardiometabolic risk factor [1].

In addition, fatty liver index (FLI) was calculated using the following expression [18]:

FLI = [e0.953 × ln TG + 0.139 BMI + 0.718 ln (GGT) + 0.053 WC − 15.745]/(1 + e0.953 ln TG + 0.139 BMI + 0.718 ln (GGT) + 0.053 WC − 15.745) × 100,

where TG stands for triglycerides and GGT is an acronym for gamma-glutamyl transferase.

We used the cutoff value of FLI ≥ 60 to rule out fatty liver disease (positive likelihood ratio = 4.3) [18].

Concomitant liver disease was ruled out by serological testing for hepatitis B and hepatitis C viral infections, serum γ-globulin and immunoglobulin G levels, as well as serum ferritin concentration.

### 2.3. Ultrasound Imaging of Rectus Femoris Muscle

The US imaging of the RFM in the dominant thigh was performed in supine position with both legs in passive extension after 10 min of rest. A 4 to 18 MHz linear probe (Philips Affinity 70) was placed perpendicular to the major axis of the dominant limb, at half of the distance between the anterior superior iliac spine and the superior border of the patella. Two measurements of the RFM were taken. US images were obtained without compression, using an excess amount of contact gel. One operator with two years of experience in muscle ultrasonography performed all examinations. After freezing the US image, the anteroposterior diameter (APD) of the RFM was measured. Semi-quantitative assessment of the RFM was performed using the Heckmatt scale [19]. According to this scale, echogenicity of normal muscle is 1. Increased echogenicity is attributed to myosteatosis and/or skeletal muscle fibrosis [15,20]. To minimize the possible adverse impact of dynamic range, gray map, line density, persistence, and IClear settings on RFM quality assessment, we conducted a US RFM study with the values of the above parameters set in the middle of their ranges [21]. RFM stiffness in the dominant thigh was measured using point-shear wave elastography. At least ten valid measurements were included in the final report. There is currently no consensus on the methodology for measuring muscle stiffness. Consequently, in our study, similarly to liver stiffness assessment, we considered muscle stiffness assessment reliable if the following criteria were met: 10 valid measurements, success rate > 60%, and the ratio of interquartile range to median (IQR/M) ≤ 30% [22,23].

### 2.4. Assessment of Body Composition

In order to assess body composition, we employed two different tools: DEXA and BIA. The following parameters of DEXA were recorded: skeletal muscle mass (SMM), appendicular skeletal muscle mass (ASMM; i.e., SMM from both legs and arms), fat mass (FM), including visceral and subcutaneous FM, body fat percentage (BFP), and also lumbar spine and femoral neck bone mineral density (BMD). According to the definition by the World Health Organization, the diagnosis of osteopenia was established when T-score values were from −1.0 to −2.5, while a T-score of −2.5 and below was a diagnostic criterion for osteoporosis [24]. In order to verify the presence of reduced SMM, we calculated two indices based on DEXA results:

(1) Appendicular skeletal muscle mass index (ASMI) is the sum of the lean muscle mass of the upper and lower limbs adjusted for the height of a patient. The threshold values of ≤7.26 kg/m^2^ for males and ≤5.50 kg/m^2^ for females were used to diagnose a reduced SMM [12].
(2) Appendicular lean mass to body weight ratio (ALM/W) was calculated as the sum of the lean muscle mass of the upper and lower limbs adjusted for the weight of a patient. The threshold values of <28.27% for males and <23.47% for females were used to diagnose a reduced SMM [10].

From BIA results, we obtained the following parameters: fat-free mass (FFM), FM, BFP, SMM, and body cell mass defined as the total mass of all cellular elements in the body that constitute all metabolically active tissues of the body, i.e., tissues involved in oxygen consumption and nutritional status) percent (BCMP). In order to verify the presence of decreased SMM, we calculated the SMM to body weight ratio (SMM/W) as SMM measured by BIA divided by weight. The threshold values of <38.2% for males and <32.2% for females were used to diagnose a reduced SMM [10].

### 2.5. Assessment of Muscle Strength and Physical Activity

Deterioration in muscle quality due to myosteatosis or fibrosis may be present long before the decline in SMM. Because US examination of skeletal muscles yields good results in diagnosing various neuromuscular diseases known to be accompanied by changes in the appearance of skeletal muscles (i.e., amplified echogenicity, decreased thickness, increased vascularization, changes in muscle architecture, etc.), we decided to assess muscle strength in addition to assessing muscle mass in the present study to elucidate the correlation between changes in muscle strength and values of RFM parameters based on US imaging. In order to assess the muscle strength of the arms and legs, we measured the GS and conducted the CST (sit-to-stand motion repeated 5 times) [25,26]. The GS on the patient’s dominant hand was determined using a hand-held mechanical dynamometer (DK-50, NTMIZ CJSC, Nizhny Tagil, Russia). Patients were asked to squeeze the dynamometer three times as hard as possible. A sixty-second rest interval was allowed before each trial. Threshold values for diagnosing decreased GS in males and females depending on their BMI values are presented in Table 1 [25].

To assess physical activity, we used the Short Form of the International Physical Activity Questionnaire (IPAQ), which is a publicly available, well-developed, and widely used instrument for assessing physical activity [27]. The questions help analyze the amount of time a subject has spent in physical activity over the past 7 days and record a patient’s activity at four levels of intensity: (1) vigorous (such as aerobics), (2) moderate (such as leisure cycling), (3) walking, and (4) sitting. The criterion for physical inactivity according to the questionnaire was less than 21 points for the group of patients 18–39 years of age, less than 14 points for the age group ranging 40–65 years, and less than 7 points for the age group over 65 years [28,29].

During CST, patients were asked to acquire the posture with their arms folded across chest and sit with their feet flat on the floor, then stand up with arms folded across chest. To complete the test, patients were asked to stand up 5 times. The time required to perform this test was recorded in seconds, and the results were scored as described in Table 2 [26].

### 2.6. Statistical Analysis

The normality of data distribution was checked using the Kolmogorov–Smirnov test. Due to non-normally distributed data, nonparametric tests were performed. Continuous data are expressed as medians and interquartile ranges (IQR), whereas qualitative variables are presented as the number of events (%). Correlations between US data, body composition parameters, muscle strength, and BMD were assessed using Spearman’s rank correlation coefficient, ρ. We used the partial correlation method to exclude the influence of a third variable (specifically, IPAQ scores, and the presence of type 2 diabetes mellitus or hypertension) on the observed correlation between US imaging findings and body composition parameters. For multiple comparisons, the nonparametric Kruskal–Wallis test was used. Comparisons between two groups were made using the Mann–Whitney U test. Results with two-sided *p* ≤0.05 were considered statistically significant. Data were analyzed using IBM SPSS statistical software version 28.0 (IBM Corp., Armonk, NY, USA).

## 3. Results

### 3.1. Patient Characteristics

A total of 90 MASLD patients, who sought medical care at the Clinical and Diagnostic Division during July–November 2023, were screened for their potential inclusion in this study. Of those, 23 patients were excluded from the final analysis for the following reasons: positive result of the test for anti-HCV antibodies (2 newly diagnosed patients); excessive alcohol consumption (3 patients with an AUDIT score ≥ 8 points); due to morbid obesity (10 patients); malignant neoplasms in anamnesis (*n* = 4: 2 patients with breast cancer, 1 patient with colon cancer, and 1 patient with kidney cancer); lower limb fractures within 2 and 3 months before the inclusion in the study (2 patients); and failure to come to the center for a scheduled visit and reply to subsequent phone calls and e-mail messages (*n* = 1). Hence, the final analysis was conducted on 67 patients who have met the requirements. Their main characteristics are listed in Table 3. The median age of patients was 58 years (IQR, 51–65); 52 (65.7%) patients were obese; only 2 patients (2.9%, both were females) had normal BMI values; however, their WC was >80 cm (91 and 94 cm, respectively), indicating the presence of cardiometabolic risks. Overall, the profile of included patients was consistent with the metabolic disorders (such as median fasting blood glucose level ≥100 mg/dL, as well as WC >94 cm in males and >80 cm in females) considered cardiometabolic risk factors characteristic of MASLD. Moreover, the presence of at least one of such factors is necessary to establish the diagnosis of MASLD according to the recently published criteria [1].

All included patients had arterial hypertension (AH) of stage 1 (*n* = 22; 32.8%), 2 (22; *n* = 32.8%), or 3 (*n* = 23; 34.8%). Type 2 diabetes mellites (T2D) was characteristic of 7 (10.4%) patients, while 9 (13.4%) patients exhibited elevated fasting blood glucose levels.

### 3.2. Assessment of Body Composition, Muscle Strength and Level of Physical Activity

Results of body composition assessment using DEXA and BIA, as well as data on muscle strength measurements in 67 MASLD patients, are presented in Table 4. Based on both DEXA and BIA results, all patients had excess absolute (kg) and relative (%) FM, amounting to >25% of body weight in men and >31% of body weight in women. It is worth noting that the amount of visceral fat in patients with MASLD was as high as the mass of subcutaneous fat, indicating the elevated risk of metabolic disorders and chronic noncommunicable diseases in these patients. Hence, we concluded that all MASLD patients included in our study were overweight or obese, regardless of the fact that two of them had normal BMI values.

As for the indices assessing SMM, ASMI was within the range of reference values both in males and females, whereas 5 men (20.8%) and 16 women (37.2%) exhibited reduced ALM/W index values. Based on BIA results, 3 males (12.5%) and 3 females (6.9%) had reduced SMM/W index values. Male and female MASLD patients with reduced ALM/W and SMM/W values did not differ statistically significantly from male and female MASLD patients with normal ALM/W and SMM/W values in terms of their age, presence of T2D, stage of AH, GS, or CST score.

**Figure 1:**
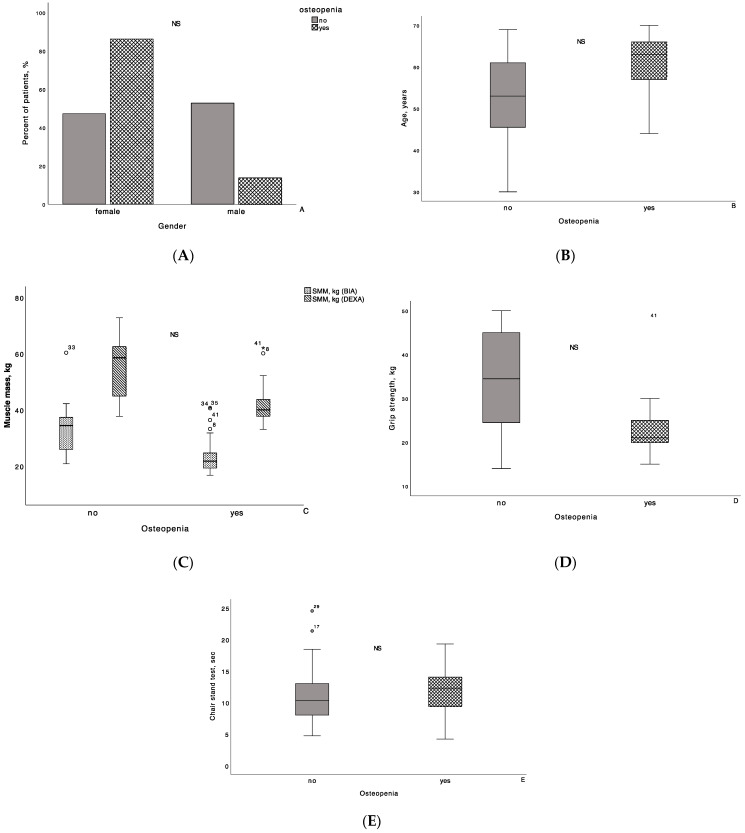
The distribution of patients with and without osteopenia by gender, age, skeletal muscle mass (SMM) and muscle strength. Patients with osteopenia did not differ significantly from patients without osteopenia in terms of their gender (**A**), age (**B**), SMM (**C**), and muscle strength (**D**,**E**). The line through the middle of each box represents the median. The length of the box, thus, represents the interquartile range. The error bars show the minimum and maximum values of each subscale. Outliers are depicted as circles and asterixis. All comparisons are performed using Mann–Whitney U test. BIA: bioelectrical impedance analysis; DEXA: dual-energy X-ray absorptiometry; NS: non-significant.

The distribution of patients with and without osteopenia by gender, age, SMM, and muscle strength is shown in Figure 1. Of two patients with osteoporosis, one was male and one was female, aged 53 and 67 years, respectively. GS value was decreased only in female patients, whereas both patients had reduced muscle mass judging from the values of the ALM/W index.

Twelve (17.6%) patients (9 of them were women; median age 52 years, IQR: 40–60.5 years) had hypodynamia according to their IPAQ scores. MASLD patients with and without hypodynamia did not differ from each other by their body composition parameters or muscle US imaging results, as well as by the presence of impaired fasting glucose levels or AH of any stage. However, T2D was significantly more common in MASLD patients with physical inactivity (*n* = 11/12 vs. *n* = 5/55, *p* = 0.014).

There was a statistically significant weak positive correlation between the IPAQ scores and ASMI (ρ = 0.264, *p* = 0.035) and a statistically significant weak negative correlation between the IPAQ scores and time required to arise from chair for 5 times (ρ = −0.250, *p* = 0.045). These correlations remained significant after the adjustment for the presence of T2D, impaired fasting glucose, or AH of any stage.

### 3.3. Sonographic Features of Rectus Femoris Muscle and Their Correlation with Body Composition Parameters

**Figure 2:**
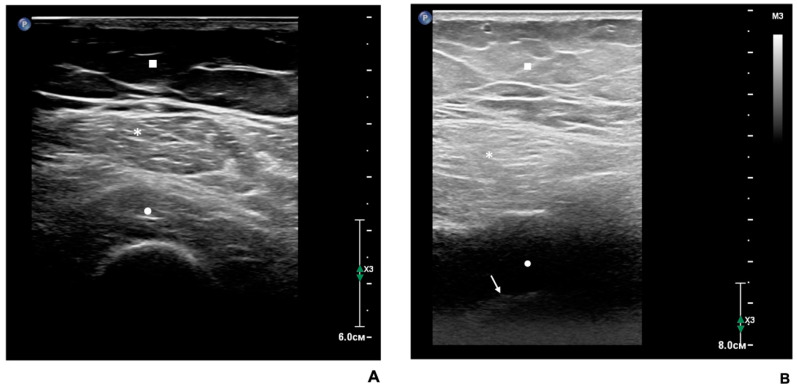
US images of the rectus femoris muscle (RFM) in patients with MASLD. (**A**) US image of the RFM of a female MASLD patient aged 68 years (body mass index = 24.7 kg/m^2^). RFM echo intensity suggests Heckmatt grade II, which implies increased muscle gray-scale level with still distinct bone echo. (**B**) US image of the RFM in a female MASLD patient aged 53 years (body mass index = 33 kg/m^2^). RFM echo intensity corresponds to Heckmatt grades III–IV, which suggests a marked increase in muscle gray-scale level with decreased bone echo (III) or complete loss of bone echo (IV). Square: subcutaneous fat; asterisks: RFM; circle: vastus intermedius. The arrow indicates the bone echo in (**B**).

Table 5 summarizes the US imaging results of RFM in the dominant hip of study participants. Visual assessment of RFM by US in most patients corresponded to grade II according to the Hekmatt grading scale [19]. Similar numbers of patients (13 and 14) were classified as grade I or grades III–IV, respectively, on the Hekmatt grading scale. Patients with grades III and IV were pooled together due to the small number of patients with grade IV echo intensity. It should be noted that patients with grades III–IV RFM echo intensity had the highest BMI and BFP values vs. the patients with echogenicity grades I or II on the Heckmatt grading scale. However, patients with echogenicity grades I, II, or III-IV on the Hekmatt scale did not differ significantly from each other in age, gender, BMI, SMM, muscle strength, FLI values, presence of T2D, impaired fasting glucose levels, AH, and its stage. Ultrasound images of the RFM in patients with MASLD included in the present study are shown in Figure 2.

We observed statistically significant moderate-to-strong direct correlations of subcutaneous fat thickness in the dominant thigh determined by US imaging and FM and BFP values measured by BIA (Spearman’s rank correlation coefficient, ρ = 0.507, *p* < 0.001; and ρ = 0.740, *p* < 0.001, respectively), as well as with the subcutaneous fat mass and BFP measured by DEXA (ρ = 0.575, *p* < 0.001 and ρ = 0.927, *p* < 0.001, respectively). However, there were no significant correlations between the visceral FM measured by DEXA and the subcutaneous fat thickness in the dominant thigh identified by US imaging. At the same time, the subcutaneous fat thickness in the dominant thigh determined by US imaging inversely correlated (moderate correlation) with the muscle mass and muscle mass indices based on both BIA and DEXA (Table 6), as well as with BCMP (weak correlation, ρ = −0.415, *p* = 0.001). These correlations remained significant after the adjustment for the presence of T2D, impaired fasting glucose level, or AH of any stage.

We observed a statistically significant weak direct correlation of the RFM APD in the dominant thigh determined via US imaging with BCMP (ρ = 0.452, *p* < 0.001) measured by BIA. We also noted a significant weak inverse correlation of the RFM APD in the dominant thigh with the BFP value measured by DEXA (ρ = −0.374, *p* = 0.003). Also, the APD of the RFM in the dominant thigh determined by US imaging directly correlated with muscle mass and muscle mass indices determined by both BIA and DEXA (weak-to-moderate correlations, Table 7).

The stiffness of RFM in the dominant thigh directly correlated with muscle mass and muscle mass indices: SMM (measured via BIA) (ρ = 0.387, *p* = 0.003; weak correlation), SMM/W (ρ = 0.350, *p* = 0.007; weak correlation), SMM (via DEXA) (ρ = 0.437, *p* = 0.001; weak correlation), ASMM (ρ = 0.461, *p* < 0.001; weak correlation), ASMI (ρ = 0.302, *p* = 0.021; weak correlation), and ALM/W (ρ = 0.277, *p* = 0.037; weak correlation). RFM stiffness exhibited an inverse correlation with BFP (measured via BIA: ρ = −0.268, *p* = 0.04; via DEXA: ρ = −0.381, *p* = 0.003; weak correlations). 

The subcutaneous fat thickness in the dominant thigh was the only US imaging parameter significantly different between the groups of MASLD patients with normal and reduced ALM/W index values (male patients, ALM/W < 28.27%: *p* = 0.001; female patients, ALM/W < 23.47%: *p* < 0.001).

**Figure 3:**
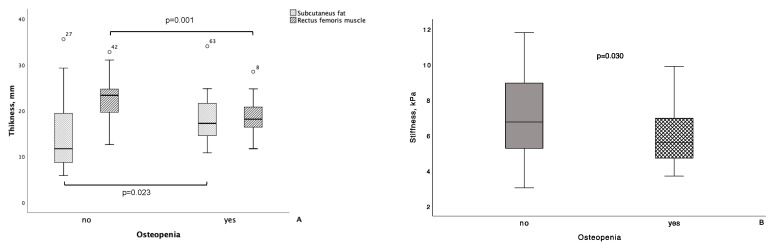
Subcutaneous fat thickness, anteroposterior diameter (APD) of the RFM, and stiffness of the RFM in the dominant thigh in patients with and without osteopenia. The line through the middle of each box represents the median. The length of the box, thus, represents the interquartile range. The error bars show the minimum and maximum values of each subscale. Outliers are depicted as circles. All comparisons are carried out using Mann–Whitney U test.

Interestingly, we observed a statistically significant weak direct correlation of the RFM APD and the stiffness of RFM in the dominant thigh with the lumbar spine BMD values (ρ = 0.350, *p* = 0.005 and ρ = 0.362, *p* = 0.005, respectively). In addition, the lumbar spine BMD values inversely correlated with the subcutaneous fat thickness in the dominant thigh (ρ = −0.259, *p* = 0.042; weak correlation). These correlations remained statistically significant even after adjustment for the presence of T2D, impaired fasting glucose level, or AH of any stage. The presence of osteopenia was associated with greater subcutaneous fat thickness in the dominant thigh (*p* = 0.023), smaller APD of the RFM (*p* = 0.001), and lower stiffness of the latter (*p* = 0.030) (Figure 3).

As for the muscle strength measurements, we obtained the following results: the time required to rise from a chair five times directly correlated with the subcutaneous fat thickness in the dominant thigh (ρ = 0.391, *p* = 0.002; weak correlation) and inversely correlated with the APD of the RFM in it (ρ = −0.336, *p* = 0.007; weak correlation). GS inversely correlated with the subcutaneous fat thickness in the dominant thigh (ρ = −0.678, *p* < 0.001; moderate correlation) and directly correlated with the RFM APD in it (ρ = 0.566, *p* < 0.001; moderate correlation). It is worth noting that both lumbar spine and femoral neck BMD values directly correlated with GS (ρ = 0.367, *p* = 0.003 and ρ = 0.454, *p* < 0.001, respectively; weak correlations). These correlations remained statistically significant even after adjustment for the presence of T2D, impaired fasting glucose level, or AH of any stage. Patients with higher CST values had greater values of subcutaneous fat thickness in the dominant thigh (*p* = 0.011) and smaller APD of the RFM (*p* = 0.040). At the same time, MASLD patients with sustained and reduced GS values differed from each other in the thickness of subcutaneous fat tissue only in the dominant thigh (*p* = 0.003). In addition, GS values were substantially lower in patients with osteopenia (*p* < 0.001).

## 4. Discussion

In our study, we used US imaging to assess the quantitative and qualitative characteristics of the RFM in the dominant thigh in order to identify their correlation with body composition parameters measured via BIA and DEXA and also with reduced muscle strength and BMD values in a group of patients with MASLD without significant liver fibrosis. 

According to our results, the majority of patients with MASLD included in the present study had grade II RFM echogenicity based on Heckmatt grading scale, which is characterized by an increased muscle gray-scale level with still clear bone echo [19]. All patients in our study population had excess BFP. Accordingly, the above-mentioned increase in echogenicity of the RFM in overweight or obese patients may be explained by increased intramuscular fat content (i.e., myosteatosis) [20]. This visual effect is clear even in the presence of increased thickness of subcutaneous fat. However, due to their greater muscle mass and thicker muscle fibers, the overall echogenicity of the RFM in adult men can be slightly lower since there are fewer tissue transitions per surface area [9]. In our study, 13 patients exhibited grade I and 14 patients grade III–IV echogenicity on the Hekmatt scale. These patients did not differ significantly from patients with grade II echogenicity in age, gender, BMI, SMM, muscle strength, or FLI index values. However, patients with grades III–IV echogenicity on the Heckmatt grading scale, characterized by greater attenuation of the acoustic signal due to an increase in the content of subcutaneous and intramuscular fat, had higher BMI values vs. patients with grades I and II echo signal intensity [20].

Our data correspond to the results of a study by G. Tarantino et al. [30], who performed the US investigation of the biceps brachii of the left superior arm in patients with MASLD and used Heckmatt scores (I–IV) to assess the degree of intramuscular triglyceride content in this muscle. The authors concluded that intramuscular triglyceride content in the biceps brachii (i.e., Heckmatt scores I, II, III, or IV) in patients with MASLD was predicted by hepatic steatosis severity determined via ultrasound investigation.

According to the published evidence, high echo intensity of skeletal muscles can be associated not only with their fatty infiltration but also with interstitial muscle fibrosis, albeit the contribution of any of these pathological changes to increased muscle echogenicity may be difficult to ascertain in an individual patient. However, using echo intensity measurements, morphometry, and biochemical tests, Reimers et al. demonstrated that adipose tissue replacement correlated better with increased muscle echogenicity than with fibrosis [31]. Thus, because the patients included in our study had no known muscle disease, were less than 70 years of age, and had excess body fat, the increased echo intensity of the RFM in patients with MASLD was probably caused by fatty infiltration rather than fibrosis.

It is worth noting that visual assessment of muscle echogenicity using the Heckmatt scale is highly subjective, and the reported sensitivity of this method is about 71–76% [20,32]. It appears that the use of quantitative measurements of skeletal muscle echo intensity may be more effective for qualitative analysis of the RFM in patients with MASLD and obesity, since this method has better diagnostic performance (higher sensitivity and higher interobserver agreement) in identifying neuromuscular disorders in children [14]. However, in our study, we did not perform quantitative gray-scale echo intensity measurements due to the lack of necessary software but rather assessed the echo intensity of the RFM in the dominant thigh using a semiquantitative Heckmatt grading scale.

A. Scafoglieri et al. assessed the intensity of the echo signal of the rectus femoris, gracilis, and rectus abdominis muscles. They established that the modification of the dynamic range, gray map, line density, persistence, and IClear settings can affect the assessment of skeletal muscle quality due to the significant influence of these parameters on the echo signal intensity of the muscles. The authors concluded that when assessing the quality of skeletal muscles, parameter settings should be fixed in their midranges in order to minimize the influence of setting-dependent factors on echo signal intensity values [21]. Therefore, to counterbalance these possible adverse effects in our study, we performed the US examination of the RFM with the settings of the above-mentioned parameters fixed within their median values. 

Overall, the results of the US imaging of the RFM in the dominant thigh in patients with MASLD showed that the greater was the subcutaneous fat thickness, the smaller was the SMM, and the lower was the muscle strength both in arms and legs. On the contrary, the greater was the APD of the RFM, the larger was the SMM, and the greater was the muscle strength both in arms and legs. Despite relatively strong correlation implying a strong association between the US imaging results and body composition parameters, only one US parameter (viz., subcutaneous fat thickness in the dominant thigh) differed significantly between MASLD patients with normal and reduced muscle mass, as well as between MASLD patients with sustained and reduced muscle strength. These data indicate that the amount of body fat per se, independently of muscle mass, may have a negative effect on the muscle quality and muscle mass, and this can be revealed during the visual assessment of muscles in the form of an increase in muscle echo intensity. In support of these findings, we confirmed that BCMP, measured by BIA and representing all metabolically active tissues of the body, correlates directly with muscle mass and inversely with the amount of subcutaneous fat measured during US examination of the dominant thigh.

The association between the fatty infiltration of skeletal muscles and progressive forms of MASLD was already described in a study by M. Nachit et al. [3], who assessed SMM and density (as a measure of fatty infiltration of the muscles) using an abdominal CT scan at the level of the L4 vertebra. They established that muscle fat content was significantly higher in patients with MASH vs. patients with simple steatosis. They also revealed that a substantial reduction in muscle fat content as a consequence of bariatric surgery or dieting significantly correlated with regression of MASH. Unfortunately, in our study, we did not perform liver biopsy to assess the severity of liver disease because the MASLD patients in the study population did not have any indication for this procedure (viz., high liver stiffness values or persistently high transaminase activity levels). However, we compared activity levels of alanine aminotransferase (ALT), which is a surrogate marker of liver disease activity [33], between MASLD patients with grade I, II, or III–IV echogenicity on the Heckmatt grading scale. Despite the lack of statistically significant differences between groups, the highest ALT activity was determined in patients with grades III–IV echo intensity of the RFM, who also had the highest BMI values among the included patients with MASLD. Assessing the relationship between sonographic features of the RFM and liver disease activity was beyond the scope of this scientific study and requires further research. 

As for the dominant hip RFM stiffness, this parameter was also inversely related to the amount of adipose tissue in the body. In their study, CJ Burke et al. assessed changes in shear wave velocity in the pronator quadratus muscle in patients after volar locking plate fixation for distal radius fractures (*n* = 17) compared with the contralateral uninjured and nonoperated side [34]. The authors found a statistically significant reduction in long-axis velocities of the pronator quadratus muscle on the treated side compared with the untreated side, which may be explained by atrophy and fatty infiltration of the pronator quadratus muscle due to disuse after injury. Similar data were obtained by AB Rosskopf et al., indicating that the shear wave velocity of the supraspinatus muscle in 22 asymptomatic middle-aged volunteers decreased with increasing fatty infiltration of the muscle [35]. Therefore, the decrease in RFM stiffness with increasing body weight and body fat content in our study may also be attributed to fatty infiltration of the muscle. In support of this assumption, it should be noted that the lowest stiffness of the RFM and, consequently, the lowest shear wave velocity along the long axis of the RFM were observed in our study in MASLD patients with grades III–IV muscle echogenicity on the Heckmatt scale (*p* = 0.097). 

We also established that both APD and stiffness of the RFM in the dominant thigh directly correlated with the lumbar spine BMD values. The association between muscle mass and BMD has already been described in several publications [36,37,38]. In fact, it was shown that the greater the muscle mass and/or strength, the higher was the BMD, and the lower was the prevalence of osteoporosis. Such a relationship between skeletal muscles and bones may be explained by the fact that static and dynamic forces generated by muscle contractions in cases of sustained muscle strength, as well as mechanical load applied to bones due to the healthy percentage of muscle mass, may exert stress on the bones, promoting bone formation and retention [39,40]. In our study, we demonstrated that US imaging parameters of the RFM characterizing muscle amount (APD) and quality (fatty infiltration of the muscle) were associated with lumbar spine BMD values. It should be noted that the latter dependence was not previously shown by other authors.

The lack of association between the femoral neck BMD values and US parameters of the RFM in our study may be explained by the earlier described lumbar spine and hip T-score discordance, ranging from 41.69% to 57.51% [41,42,43]. Such discordance is habitually explained by technical reasons, such as artifacts and improper patient positioning, as well as by several physiological and pathological factors. For instance, estrogen deficiency in postmenopausal women is known to accelerate trabecular bone loss [44], thereby reducing lumbar spine BMD, which may result in BMD discordance. Pathological factors involve those associated with the diseases related to aging, such as osteophytosis, vertebral sclerosis, and aortic calcification. These conditions may contribute to the overestimation of T-score of lumbar spine BMD, which yields higher lumbar spine BMD values vs. femoral neck BMD values. The other factor capable of affecting BMD values measured at different locations may be the increased BMI, in particular, due to high fat content in the body, which was the case for all MASLD patients included in our study. It has previously been shown that soft tissues may obscure the BMD determination by DEXA, leading to the BMD measurement error [45]. Accordingly, Zhu et al. demonstrated that women with high body fat, as implied by their BMI value, had significantly lower BMD values at the femoral neck [46]. The latter may be true for our study population, consisting of 64.2% of females with excess body weight and body fat.

The present study also indicates that the level of physical activity assessed via the IPAQ questionnaire correlates positively with muscle mass and negatively with time required to perform the chair stand test, a method being used to assess muscle strength in legs as well as muscle function. In accordance with these data, G. Distefano et al. have reported that a sedentary lifestyle may contribute to “unhealthy aging” [47]. This term denotes an impairment in muscle regeneration capacity and contractile function, as well as an increase in muscle fat infiltration. Taken together, these changes contribute to the mobility limitations, increased risk of falls, functional limitations, and mortality. Moreover, obesity may potentiate these effects of physical inactivity. So, we can conclude that there is a strong unfavorable association between hypodynamia, obesity, and muscle quality and quantity capable of impacting patients’ quality of life, general health, and life expectancy, and that timely assessment of skeletal muscle condition, as well as timely interventions to increase muscle mass and improve their structure and function, appear to be a key to the prevention and treatment of MASLD and possibly other chronic diseases. In addition, muscle quality and quantity assessment in patients with MASLD could constitute a promising direction in terms of using an individualized approach to developing recommendations for lifestyle modifications.

Although muscle US imaging is not currently recommended for routine clinical practice according to the Revised European Consensus on the Definition and Diagnosis of Sarcopenia [12], the diagnostic performance of this method for assessing muscle quantity and quality is widely studied in various diseases and pathological conditions, including trauma patients [48], critically ill patients [49], and patients with muscular dystrophy [14]. It is now generally accepted that the musculoskeletal system is involved in the pathogenesis of chronic diseases of various organs, such as chronic kidney disease [50], cardiovascular diseases [51], T2D and metabolic syndrome [52,53], as well as MASLD [3,7,16], due to the production of biologically active substances called myokines and a direct effect on physical performance, which is believed to be influenced by muscle mass and quality [4]. Besides that, there is no doubt that increasing physical activity is an important measure that can prevent MASLD and/or reduce the risk of its progression and complications [10,11]. This increase is possible, provided that the muscles are healthy. Thus, assessing the quality and quantity of muscle in patients with MASLD may be a promising direction in terms of improving the prognosis of patients and using an individualized therapeutic approach to develop recommendations for lifestyle modification.

Currently available methods of assessing muscle mass and quality, such as DEXA, BIA, CT, or MRI, have several disadvantages, including the lack of portability and radiation exposure (CT, DEXA). This fact limits their use and repeated testing. Consequently, studying the possibilities of using US imaging for assessing muscle condition in patients with MASLD is highly warranted.

## 5. Limitations

Our study has several limitations. First, we had a relatively small sample size, albeit the participants constituted a representative sample of well-characterized MASLD patients without significant liver fibrosis, and the sample size was compatible with that in other studies investigating the opportunities of using US examination of skeletal muscles in different categories of patients [20,21,31,34]. Also, we used solely the APD of the RFM as a surrogate measure of muscle mass, because it is easy to measure US imaging parameters. Because the patients included in our study were overweight or obese and had large thigh circumferences, we could not measure RFM diameter using a linear transducer. Another limitation is that when assessing muscle echo intensity, we used the semiquantitative Heckmatt scale with acceptable sensitivity, although the use of quantitative gray-scale echo intensity measurements may be more accurate and reliable in assessing muscle echogenicity and therefore muscle quality. The fourth limitation is that we did not carry out MRI or CT to assess the presence and extent of fatty infiltration in muscles as the reference method for the US imaging. However, we determined RFM stiffness and muscle echogenicity, which have been shown to correlate with muscle fatty infiltration. In our study, these US imaging parameters exhibited unidirectional associations with BFP. One more limitation is that in this study, we did not assess patients’ functionality with special questionnaires, and its association with body composition parameters, US characteristics of rectus femoris muscle, muscle strength, or function. This issue requires further investigation in separate studies. Finally, given the design of the study and the employed statistical methods, based on the obtained data, it is not possible to draw conclusions about the presence and nature of the cause-and-effect relationship between MASLD and US characteristics of the RFM. 

Despite these limitations, our study confirmed that US imaging parameters of the RFM measured at the dominant hip significantly correlated with muscle mass, strength, body fat, and BMD of the lumbar spine. Future studies with larger sample sizes will help identify possible threshold values for subcutaneous fat thickness and/or RFM stiffness that could predict the decreased muscle mass, strength, or BMD in patients with MASLD.

## 6. Conclusions

We conclude that US examination of RFM in the dominant thigh is a reliable and accurate method for the assessment of the musculoskeletal system in patients with MASLD. Its results correlate significantly with body composition parameters, including muscle mass and BFP measured by both DEXA and BIA, as well as with muscle strength in arms and legs and lumbar spine BMD values. Sonographic measurement of RFM quality, thickness, and stiffness provides an easy-to-use objective tool for assessing muscle quality and mass, which allows to receive immediate results and decide on the need and indications for further evaluation. Moreover, it can assist in early bedside identification of patients who are at high risk for developing reduced BMD.

## Figures and Tables

**Table 1:** Threshold values for the diagnosis of poor grip strength (GS) in males and females depending on their body mass index values (BMI) [25].

BMI, kg/m^2^	Threshold Values for Diagnosing Poor GS (kg)
Males	
≤24	≤29
24.1–26	≤30
26.1–28	≤30
>28	≤32
Females	
≤23	≤17
23.1–26	≤17.3
26.1–29	≤18
>29	≤21

**Table 2:** Scoring the repeated chair stand test (CST) [26].

CST Time, s	Points
Participant unable to complete 5 repetitions of CST or completes CST in >60 s	0
≥16.7	1
13.7–16.69	2
11.2–13.69	3
≤11.19	4

**Table 3:** Demographic, anthropometric, and biochemical parameters of 67 MASLD patients.

Parameter	MASLD Patients (*n* = 67)
Gender: female, *n* (%)	43 (64.2)
Age, years	58 (51–65)
BMI, kg/m^2^	33.2 (30.4–36.9)
Normal weight (BMI < 25 kg/m^2^), *n* (%)	2 (2.9)
Overweight (25 ≤ BMI < 30 kg/m^2^), *n* (%)	13 (19.4)
Obese (BMI ≥ 30 kg/m^2^), *n* (%)	52 (77.6)
Waist circumference (males + females), cm Waist circumference (males), cm Waist circumference (females), cm	106.5 (102–113.2) 108 (106–116.5) 106 (100–112.3)
Hip circumference, cm	114 (109–119)
Platelet count, 10^9^/L	245 (210–286)
WBC, 10^9^/L	6.1 (5.3–7.2)
ALT, IU/L	23 (17–38)
AST, IU/L	22 (18–26)
GGT, IU/L	31.5 (21–46.3)
Total bilirubin, mg/dL	0.7 (0.53–0.88)
Glucose, mg/dL	102.6 (97.2–109.8)
Total cholesterol, mg/dL	223.1 (196.2–238.5)
Triglycerides, mg/dL	126.6 (91.8–180.9)
CRP, mg/L	2.7 (1.4–5.5)
Creatinine, μmol/L	74 (68–88)
Uric acid, μmol/L	357 (315.4–416.5)
Insulin, μIU/mL	10.7 (8.5–14.3)
HOMA-IR > 2.7, *n* (%)	47 (70.1)
FLI	84.5 (70.5–93.8)
FLI ≥30, but <60, *n* (%)	13 (19.4)
FLI ≥60, *n* (%)	54 (80.6)
TyG ≥ 4.49, *n* (%)	54 (80.6)
Liver stiffness, kPa (point-shear wave elastography, pSWE)	5.1 (4.1–6.3)

Note: The presented values denote frequency (%) or median (interquartile range). ALT: alanine aminotransferase; AST: aspartate aminotransferase; BMI: body mass index; CRP: C-reactive protein; FLI: fatty liver index; GGT: gamma-glutamyl transferase; HOMA-IR: homeostasis model assessment of insulin resistance; TyG: triglyceride glucose index; WBC: white blood cell count.

**Table 4:** Parameters of body composition and measurements of muscle strength.

Parameter	Value
Dual-energy X-ray absorptiometry	
SMM, g	44,548 (39,052–59,651)
ASMM, g	22,525 (19,909–31,061)
Visceral FM, g	2127.5 (1500.8–2616)
Subcutaneous FM, kg	2.382 (1730.5–2907.3)
BFP, %	43.5 (36.2–47.9)
Lumbar spine BMD, g/cm^2^	1.22 (1.1–1.35)
Femoral neck BMD, g/cm^2^	0.98 (0.85–1.04)
ASMI, kg/m^2^: males	9.97 (8.9–10.7)
ASMI, kg/m^2^: females	7.89 (7.3–8.6)
ALM/W, %: males	30 (29.1–32.01)
ALM/W, %: females	23.9 (22.3–24.95)
Osteopenia, *n* (%)	29 (43.3)
Osteoporosis, *n* (%)	2 (2.98)
Bioimpedance analysis	
FFM, kg	55.2 (48.3–71.5)
SMM, kg	25.5 (20.9–35.9)
SMM/W, %: males	49 (48–49.8)
SMM/W, %: females	42.3 (40.6–43.1)
FM, kg	34.5 (29.2–40.7)
BFP, %	40.4 (31.7–43.9)
BCMP, %	55.4 (52.5–58.7)
Muscle strength measurements	
GS, kg: males	42.5 (35.3–48.0)
GS, kg: females	23 (20–25)
Chair stand test, s	10.9 (8.6–13.8)
Hypodynamia according to IPAQ score, *n* (%)	12 (17.6)

Note: ALM/W: appendicular lean mass to body weight ratio; ASMI: appendicular skeletal muscle mass index; ASMM: appendicular skeletal muscle mass; BCMP: body cell mass percent; BFP: body fat percentage; FFM: fat-free mass; FM: fat mass; GS: grip strength; SMM: skeletal muscle mass; SMM/W: skeletal muscle mass to body weight ratio; IPAQ: International Physical Activity Questionnaire.

**Table 5:** Ultrasound (US) imaging parameters of rectus femoris muscle (RFM).

Parameter	Value
Subcutaneous fat thickness, mm	16.3 (10.9–21.5)
RFM thickness, mm	20.6 (17.5–24.1)
Heckmatt grading scale	
Grade I, *n* (%); [male–female]	13 (19.4); [4:9]
Grade II, *n*(%) [male–female]	40 (59.7); [15:23]
Grade III-IV, *n* (%) [male–female]	14 (20.9); [5:8]
RFM stiffness, kPa	6.4 (4.8–7.9)

**Table 6:** Correlations of subcutaneous fat thickness in the dominant thigh determined by ultrasound imaging with muscle mass and muscle mass indices identified by both BIA and DEXA.

Skeletal Muscles Parameters	Spearman’s Rank Correlation Coefficients for the Subcutaneous Fat Thickness in the Dominant Thigh (ρ) and Corresponding *p*-Values
Dual-energy X-ray absorptiometry	
SMM, g	−0.672, *p* < 0.001
ASMM, g	−0.662, *p* < 0.001
ASMI, kg/m^2^	−0.476, *p* < 0.001
ALM/W, %	−0.892, *p* < 0.001
Bioimpedance analysis	
SMM, kg	−0.545, *p* < 0.001
SMM/W, %	−0.592, *p* < 0.001

Note: ALM/W: appendicular lean mass-to-body weight ratio; ASMI: appendicular skeletal muscle mass index; ASMM: appendicular skeletal muscle mass; SMM: skeletal muscle mass; SMM/W: skeletal muscle mass-to-body weight ratio.

**Table 7:** Correlations between the anteroposterior diameter (APD) of the RFM in the dominant thigh determined by US imaging and muscle mass, as well as muscle mass indices identified by both BIA and DEXA.

Skeletal Muscle Parameters	Spearman’s Rank Correlation Coefficients for the APD of the RFM in the Dominant Thigh (ρ) and Corresponding *p*-Values
Dual-energy X-ray absorptiometry	
SMM, g	0.687, *p* < 0.001
ASMM, g	0.710, *p* < 0.001
ASMI, kg/m^2^	0.685, *p* < 0.001
ALM/W, %	0.402, *p* = 0.001
Bioimpedance analysis	
SMM, kg	0.489, *p* < 0.001
SMM/W, %	0.420, *p* = 0.001

Note: ALM/W: appendicular lean mass-to-body weight ratio; ASMI: appendicular skeletal muscle mass index; ASMM: appendicular skeletal muscle mass; SMM: skeletal muscle mass; SMM/W: skeletal muscle mass-to-body weight ratio.

## Data Availability

The data presented in this study are available on reasonable request from the corresponding author. The data are not publicly available due to privacy restrictions.
